# Supplementary material for: Home hazard modification programs for reducing falls in older adults: a systematic review and meta-analysis
Source: PeerJ. 2023 Jul 20;11:e15699. doi: 10.7717/peerj.15699 (PMC10363339; doi:10.7717/peerj.15699)
Supplement: Supplemental Information 2 [file peerj-11-15699-s002.docx]

**The rationale for conducting this systematic review meta-analysis**

Environmental factors are the major cause of falls in older adults, particularly falls occurring at homes (Zhang et al., 2019; Sophonratanapokin, Sawangdee & Soonthorndhada, 2012). Home modification is a potential intervention to reduce fall risks in this population (Hill et al., 2018). The term “home modification” is used to describe the act of changing settings to reduce the number of accidents and promote independent living (McCullagh, 2006). Previous systematic reviews show the significant results of evidence that identified for both single- and multi-component treatments that included home modifications to reduce the number and risk of falls among older adults and to improve function for people with a variety of health conditions. (Stark et al., 2017). Stark et al., 2021 constructed randomized clinical trial, showed that among community-dwelling older adults who were at high risk for falling, a brief program to remove home hazards did not lower the risk of falls. A specified secondary outcome of the intervention, a lower rate of falls, was achieved. Additionally, another systematic review reveals the positive outcomes of home modification interventions in enhancing participation for community-dwelling individuals and older adults (Chase et al., 2012). A Meta-Analysis of Randomized Trials on environmental interventions to prevent falls in community-dwelling older people showed a 21% decrease in the incidence of falls (relative risk [RR] = 0.79; 0.65 to 0.97). The significant treatment impact of one trial was the cause of the heterogeneity. Analysis of four trials showed a clinically significant 39% reduction in falls (RR = 0.61; 0.47 to 0.79), representing an absolute risk difference of 26% for a number needed to treat four individuals (Clemson et al., 2008).

Recent evidence suggests that home modification can enhance the performance and safety of older adults with functional impairments. Nevertheless, previous evidence showed some ambiguous results. A meta-analysis of current scientific evidence would be beneficial for fulfilling this knowledge gap of the effectiveness of home modification interventions. Thus, the goal of this systematic review and meta-analysis was to update prior reviews, investigate the evidence, and characterize the effectiveness of the evidence for the usefulness of environmental interventions in preventing falls, as well as to improve the precision of known results.
