# Supplementary material for: Home hazard modification programs for reducing falls in older adults: a systematic review and meta-analysis
Source: PeerJ. 2023 Jul 20;11:e15699. doi: 10.7717/peerj.15699 (PMC10363339; doi:10.7717/peerj.15699)
Supplement: Supplemental Information 3 [file peerj-11-15699-s003.docx]

**Supplementary Material**

**Supplementary Table S1.** Full search strategy: search strategy example (MEDLINE 1999 to
January 2023)

|  | **Keywords** | **Combination** |
| --- | --- | --- |
| Population | 1. (exp older people$/ or exp elder$/ or exp older adult$/ or exp age$/ or exp senior$/ or exp frail/) adj3 elderly/ 2. exp geriatrics/ or exp older person$/ or exp older population$/ 3. (older subject$ or older patient$ or older age$ or older adult$ or older man or older men or older male$ or older woman or older women or older female$ or older population$).mp. or older person$.tw. | 1. 1 or 2 or 3 |
| Intervention | 1. (home safety or environment modification or housing improvement or person-environment fit).mp. 2. (environment design$ or person environment$ or housing adap?tion$).mp. 3. home hazard$/ or home modification$/ or home adaptation$/ or home environment$/ | 1. 5 or 6 or 7 |
| Studies | 1. Randomized Controlled Trials.tw. 2. random allocation/ 3. Controlled Clinical Trial$.tw. 4. control group$/ 5. clinical trials.tw. 6. double-blind method$/ or single-blind method$.mp 7. (random$ or RCT or RCTs).tw. 8. (controlled adj5 (trial$ or stud$)).tw. 9. (clinical$ adj5 trial$).tw. 10. ((control or treatment or experiment$ or intervention) adj5 (group$ or subject$ or patient$)).tw. 11. ((singl$ or doubl$ or tripl$ or trebl$) adj5 (blind$ or mask$)).tw. 12. (assign$ or allocat$).tw. 13. controls.tw. | 1. 9 or 10 or 11 or 12 or 13 or 14 or 15 or 16 or 17 or 18 or 19 or 20 or 21 |
| Outcome | 1. (risk of fall$ or fall$).mp. 2. exp Accidental Falls/ 3. preventing fall$.mp. 4. fall$ prevention.mp. 5. exp Accidents, Home/mo, pc [Mortality, Prevention & Control] | 1. 23 or 24 or 25 or 26 or 27 |
|  | 1. effectiveness.tw. 2. (evaluation or program$ evaluation).tw. 3. assessment.mp. or assess$.tw. 4. efficacy.mp. or efficiency.tw. | 1. 29 or 30 or 31 or 32 2. 4 and 8 and 22 and 28 and 33 |

**Supplementary Table S2. Detail of the papers excluded after full text review.**

| **Reason for exclusion** | **study** |
| --- | --- |
| Not reported falling as primary outcome | 1. Greiman L, et al. 2022 2. Nguyen AT, et al. 2020 3. Wilson N, et al. 2017 4. M. D. Keall, et al. 2017 5. Pega F, et al. 2016 6. Wyman JF, et al. 2007 7. Gitlin LN, et al. 2006 8. Sweeney MA and Chiriboga DA. 2003 |
| Not randomized controlled trials | 1. Ahmad Ainuddin H et al, 2023 2. Greiman L, et al. 2022 3. Nguyen AT, et al. 2020 |
| Home modification program < 75% | 1. Pip A Logan, et al. 2021 2. Tan PJ, et al. 2018 3. M. Palvanen, et al. 2013 4. M. R. Hendriks, et al. 2008 5. N. M. Sjosten, et al. 2007 6. M. R. Hendriks, et al. 2005 7. R. G. C. L. Clemson, et al. 2004 8. E. Lightbody, et al. 2002 9. L. Day, et al. 2002 10. N. Peel, et al. 2000 |
| Not related to aging population | 1. M. D. Keall, et al. 2021 2. M. D. Keall, et al. 2015 |
| No full paper in English version | 1. Jo, et al. 2018 |

**References to studies excluded from this review.**

1. Greiman L, Koon L, Schulz JA, Nary D. A usable home: A qualitative investigation of the relationship between home usability and community participation for people with disabilities. Disabil Health J. 2022;15(1): 1-7. <https://doi.org/10.1016/j.dhjo.2021.10121>.
2. Nguyen AT, Somerville EK, Espín-Tello SM, Keglovits M, Stark SL. A Mobile App Directory of Occupational Therapists Who Provide Home Modifications: Development and Preliminary Usability Evaluation. *JMIR Rehabil Assist Technol*. 2020;7(1):e14465. Published 2020 Mar 30. doi:10.2196/14465.
3. Wilson N, Kvizhinadze G, Pega F, Nair N, Blakely T. Home modification to reduce falls at a health district level: Modeling health gain, health inequalities and health costs. PLoS One. 2017;12(9):e0184538. Published 2017 Sep 14. doi:10.1371/journal.pone.0184538.
4. Keall MD, Pierse N, Howden-Chapman P, Guria J, Cunningham CW, Baker MG. Cost-benefit analysis of fall injuries prevented by a programme of home modifications: a cluster randomised controlled trial. Inj Prev. 2017;23(1):22-26. doi:10.1136/injuryprev-2015-041947
5. Pega F, Kvizhinadze G, Blakely T, Atkinson J, Wilson N. Home safety assessment and modification to reduce injurious falls in community-dwelling older adults: cost-utility and equity analysis. Inj Prev. 2016;22(6):420-426. doi:10.1136/injuryprev-2016-041999
6. Wyman JF, Croghan CF, Nachreiner NM, et al. Effectiveness of education and individualized counseling in reducing environmental hazards in the homes of community-dwelling older women. J Am Geriatr Soc. 2007;55(10):1548-1556. doi:10.1111/j.1532-5415.2007.01315.
7. Gitlin LN, Winter L, Dennis MP, Corcoran M, Schinfeld S, Hauck WW. A randomized trial of a multicomponent home intervention to reduce functional difficulties in older adults. J Am Geriatr Soc. 2006;54(5):809-816. doi:10.1111/j.1532-5415.2006.00703.x
8. Sweeney MA, Chiriboga DA. Evaluating the effectiveness of a multimedia program on home safety. Gerontologist. 2003;43(3):325-334. doi:10.1093/geront/43.3.325
9. Ahmad Ainuddin H, Romli MH, S. F. Salim M, Hamid TA, Mackenzie L (2023) A validity study to consult on a protocol of a home hazard management program for falls prevention among community dwelling stroke survivors. PLOS ONE 18(1): e0279657. <https://doi.org/10.1371/journal.pone.0279657>
10. Logan, P. A., Horne, J. C., Gladman, J. R. F., Gordon, A. L., Sach, T., Clark, A., Robinson, K., Armstrong, S., Stirling, S., Leighton, P., Darby, J., Allen, F., Irvine, L., Wilson, E. C. F., Fox, C., Conroy, S., Mountain, G., McCartney, K., Godfrey, M., & Sims, E. (2021). Multifactorial falls prevention programme compared with usual care in UK care homes for older people: multicentre cluster randomised controlled trial with economic evaluation. BMJ (Clinical research ed.), 375, e066991. <https://doi.org/10.1136/bmj-2021-066991>
11. Tan PJ, Khoo EM, Chinna K, et al. Individually-tailored multifactorial intervention to reduce falls in the Malaysian Falls Assessment and Intervention Trial (MyFAIT): A randomized controlled trial. PLoS One. 2018;13(8):e0199219. Published 2018 Aug 3. doi:10.1371/journal.pone.0199219
12. Palvanen M, Kannus P, Piirtola M, Niemi S, Parkkari J, Järvinen M. Effectiveness of the Chaos Falls Clinic in preventing falls and injuries of home-dwelling older adults: a randomised controlled trial. Injury. 2014;45(1):265-271. doi:10.1016/j.injury.2013.03.010
13. Hendriks MR, Bleijlevens MH, van Haastregt JC, et al. Lack of effectiveness of a multidisciplinary fall-prevention program in elderly people at risk: a randomized, controlled trial. J Am Geriatr Soc. 2008;56(8):1390-1397. doi:10.1111/j.1532-5415.2008.01803.x
14. Sjösten NM, Salonoja M, Piirtola M, et al. A multifactorial fall prevention programme in home-dwelling elderly people: a randomized-controlled trial. Public Health. 2007;121(4):308-318. doi:10.1016/j.puhe.2006.09.018
15. Hendriks MR, van Haastregt JC, Diederiks JP, Evers SM, Crebolder HF, van Eijk JT. Effectiveness and cost-effectiveness of a multidisciplinary intervention programme to prevent new falls and functional decline among elderly persons at risk: design of a replicated randomised controlled trial [ISRCTN64716113]. BMC Public Health. 2005;5:6. Published 2005 Jan 14. doi:10.1186/1471-2458-5-6
16. Clemson L, Cumming RG, Kendig H, Swann M, Heard R, Taylor K. The effectiveness of a community-based program for reducing the incidence of falls in the elderly: a randomized trial. J Am Geriatr Soc. 2004;52(9):1487-1494. doi:10.1111/j.1532-5415.2004.52411.x
17. Lightbody E, Watkins C, Leathley M, Sharma A, Lye M. Evaluation of a nurse-led falls prevention programme versus usual care: a randomized controlled trial. Age Ageing. 2002;31(3):203-210. doi:10.1093/ageing/31.3.203
18. Day L, Fildes B, Gordon I, Fitzharris M, Flamer H, Lord S. Randomised factorial trial of falls prevention among older people living in their own homes. BMJ. 2002;325(7356):128. doi:10.1136/bmj.325.7356.128
19. Peel N, Steinberg M, Williams G. Home safety assessment in the prevention of falls among older people. Aust N Z J Public Health. 2000;24(5):536-539. doi:10.1111/j.1467-842x.2000.tb00506.x
20. Keall MD, Tupara H, Pierse N, et al. Home modifications to prevent home fall injuries in houses with Māori occupants (MHIPI): a randomised controlled trial. Lancet Public Health. 2021;6(9):e631-e640. doi:10.1016/S2468-2667(21)00135-3
21. Keall MD, Pierse N, Howden-Chapman P, et al. Home modifications to reduce injuries from falls in the home injury prevention intervention (HIPI) study: a cluster-randomised controlled trial. Lancet. 2015;385(9964):231-238. doi:10.1016/S0140-6736(14)61006-0
22. Jo Y-J, Kim H. Effects of Occupation-Based Home Modification and Intervention Using Assistive Devices on Activities of Daily Living Performance and Risk Factors at Home in Community–Dwelling People With Disabilities. Journal of Korean Society of Occupational Therapy. 2018;26(3):79-90.

**Supplementary Table S3. TIDieR table.**

|  | **Brief name** | | **Why** | | **Material** | | **Procedure** | | **Who provide** | | **How** | | **Where** | | **When and what** | | **Tailoring** | | **Modification** | | **Planed** | | **Actual** | |
| --- | --- | --- | --- | --- | --- | --- | --- | --- | --- | --- | --- | --- | --- | --- | --- | --- | --- | --- | --- | --- | --- | --- | --- | --- |
| **Author** | **E** | **C** | **E** | **C** | **E** | **C** | **E** | **C** | **E** | **C** | **E** | **C** | **E** | **C** | **E** | **C** | **E** | **C** | **E** | **C** | **E** | **C** | **E** | **C** |
| Susan stark, et al.2021 | 🗹 | 🗷 | 🗹 | 🗷 | 🗹 | 🗹 | 🗹 | 🗷 | 🗷 | 🗹 | 🗹 | 🗷 | 🗹 | 🗹 | 🗹 | 🗷 | 🗷 | 🗷 | 🗷 | 🗷 | 🗹 | 🗷 | Y | 🗷 |
| Cockayne S, et al. 2021 | 🗹 | 🗹 | 🗹 | 🗷 | 🗹 | 🗹 | 🗹 | 🗷 | 🗹 | 🗹 | 🗹 | 🗷 | 🗹 | 🗹 | 🗷 | 🗷 | 🗷 | 🗷 | 🗷 | 🗷 | 🗹 | 🗷 | 🗷 | 🗷 |
| Chu MM, et al. 2017 | 🗹 | 🗷 | 🗷 | 🗷 | 🗹 | 🗹 | 🗹 | 🗹 | 🗷 | 🗹 | 🗹 | 🗹 | 🗷 | 🗷 | 🗹 | 🗷 | 🗷 | 🗷 | 🗷 | 🗷 | 🗹 | 🗷 | 🗷 | 🗷 |
| Tomoko K, et al. 2015 | 🗹 | 🗷 | 🗹 | 🗷 | 🗹 | 🗹 | 🗹 | 🗹 | 🗷 | 🗹 | 🗹 | 🗹 | 🗷 | 🗷 | 🗹 | 🗹 | 🗷 | 🗷 | 🗷 | 🗷 | 🗷 | 🗷 | 🗷 | 🗷 |
| Pighills AC, et al. 2011 | 🗷 | 🗷 | 🗹 | 🗹 | 🗹 | 🗷 | 🗹 | 🗷 | 🗹 | 🗷 | 🗹 | 🗷 | 🗷 | 🗷 | 🗹 | 🗷 | 🗷 | 🗷 | 🗷 | 🗷 | 🗹 | 🗷 | 🗹 | 🗷 |
| la Grow MRS, et al. 2006 | 🗷 | 🗷 | 🗷 | 🗷 | 🗹 | 🗹 | 🗷 | 🗷 | 🗹 | 🗹 | 🗹 | 🗹 | 🗷 | 🗷 | 🗷 | 🗷 | 🗹 | 🗷 | 🗷 | 🗷 | 🗹 | 🗷 | 🗷 | 🗷 |
| Campbell AJ, et al. 2005 | 🗷 | 🗷 | 🗷 | 🗷 | 🗹 | 🗹 | 🗷 | 🗷 | 🗹 | 🗹 | 🗹 | 🗹 | 🗷 | 🗷 | 🗷 | 🗷 | 🗹 | 🗷 | 🗷 | 🗷 | 🗹 | 🗷 | 🗷 | 🗷 |
| Nikolaus T and Bach M. 2003 | 🗹 | 🗷 | 🗹 | 🗷 | 🗹 | 🗷 | 🗹 | 🗷 | 🗹 | 🗷 | 🗹 | 🗷 | 🗹 | 🗷 | 🗷 | 🗷 | 🗹 | 🗷 | 🗷 | 🗷 | 🗷 | 🗷 | 🗷 | 🗷 |
| Pardessus V, et al. 2002 | 🗷 | 🗷 | 🗹 | 🗷 | 🗹 | 🗷 | 🗹 | 🗹 | 🗷 | 🗹 | 🗹 | 🗹 | 🗹 | 🗹 | 🗷 | 🗷 | 🗷 | 🗷 | 🗷 | 🗷 | 🗷 | 🗷 | 🗷 | 🗷 |
| Stevens M. 2001 | 🗷 | 🗷 | 🗹 | 🗷 | 🗹 | 🗹 | 🗷 | 🗷 | 🗹 | 🗷 | 🗷 | 🗷 | 🗹 | 🗹 | 🗷 | 🗷 | 🗷 | 🗷 | 🗷 | 🗷 | 🗷 | 🗷 | 🗷 | 🗷 |
| Gerson LW, et al. 2005 | 🗷 | 🗷 | 🗷 | 🗷 | 🗹 | 🗷 | 🗹 | 🗷 | 🗹 | 🗹 | 🗹 | 🗹 | 🗹 | 🗹 | 🗹 | 🗹 | 🗷 | 🗷 | 🗷 | 🗷 | 🗷 | 🗷 | 🗷 | 🗷 |
| Cumming RG, et al. 1999 | 🗷 | 🗷 | 🗹 | 🗷 | 🗹 | 🗷 | 🗹 | 🗷 | 🗹 | 🗷 | 🗹 | 🗷 | 🗷 | 🗷 | 🗷 | 🗷 | 🗷 | 🗷 | 🗷 | 🗷 | 🗹 | 🗷 | 🗷 | 🗷 |

E: Experimental group, C: Control group, 🗷 Not Achieving, 🗹 Achieving
